# Highly Effective mRNA-LNP Vaccine Against Respiratory Syncytial Virus (RSV) in Multiple Models

**DOI:** 10.3390/vaccines13060625

**Published:** 2025-06-10

**Authors:** Huarong Bai, Xueliang Yu, Yue Gao, Qin Li, Baigang Wen, Rongkuan Hu

**Affiliations:** Starna Therapeutics Co., Ltd., Suzhou 215123, China; baihuarong@starnatx.com (H.B.); gaoyue@starnatx.com (Y.G.); liqin@starnatx.com (Q.L.);

**Keywords:** respiratory vaccine, mRNA delivery, RSV, lipid nanoparticles, biomaterials

## Abstract

Background: The transmembrane fusion (F) protein of RSV plays important roles in RSV pathogenesis as it mediates the fusion between the virus and the target cell membrane. During the fusion process, the F protein transits from a metastable state (prefusion, preF) to a stable state (postfusion, postF) after the merging of the virus and cell membranes. The majority of highly neutralizing antibodies induced by natural infection or immunization target the preF form, which makes it the preferred antigen for vaccine development. Methods: Here, we designed an effective RSV mRNA vaccine, STR-V003, consisting of mRNA encoding preF protein in lipid nanoparticles (LNPs). The immunogenicity, protection efficacy and toxicity were measured in multiple animal models. Results: STR-V003 demonstrated robust immunogenicity in both mice and cotton rats, inducing high levels of neutralizing antibodies and RSV preF-specific IgG antibodies and significantly reducing the RSV viral loads in the lung and nose tissue of challenged animals. In addition, STR-V003 did not show significant enhancement of lung pathology without causing vaccine-enhanced disease (VED). The repeated dose general toxicology studies and local tolerance studies of STR-V003 were evaluated in rats and non-human primate (NHP). Conclusions: STR-V003 demonstrates a favorable safety profile and induces robust protective immunity against RSV.

## 1. Introduction

Human respiratory syncytial virus (RSV) is an enveloped, single-stranded and negative-sense RNA virus, which causes a major burden on public health, both in developing and in industrialized countries. There are two RSV sub-types, RSV A and RSV B, which both cocirculate and cause illnesses during each respiratory season [1,2]. The transmembrane fusion (F) protein of RSV plays important roles in RSV pathogenesis, as it mediates the fusion between the virus and the target cell membrane. During the fusion process, F protein transits from a metastable state (prefusion, preF) to a stable state (postfusion, postF) after the merging of the virus and cell membranes [3]. The majority of highly neutralizing antibodies induced by natural infection or immunization targets the preF form, making it the preferred antigen for vaccine development [4,5,6,7]. F protein contains antigenic determinants associated with neutralizing antibodies and cytotoxic T-lymphocyte (CTL)-mediated immunity. Some antigenic sites (II and IV) are common between both pre- and postfusion forms, while others are form-specific (e.g., V and Φ are preF specific) [1,8,9].

To date, two RSV protein subunit vaccines, which induce immune responses against RSV preF protein, were approved by the FDA for adults 60 years old and over, namely, Arexvy (developed by GSK) and Abrysvo (developed by Pfizer) [10]. The latter vaccine (Abrysvo) was also approved by the FDA for pregnant women to protect newborns and infants against severe RSV disease in the first 6 months after birth [11]. Due to efficacy and safety, rapid development capacity and the possibility for rapid manufacturing, mRNA vaccines proved to be great substitutes for traditional vaccines during the COVID-19 pandemic. Using the same RSV preF antigen, Moderna’s mRNA-based mResvia became the third RSV vaccine approved by the FDA [12]. In this study, we developed an mRNA vaccine, STR-V003, produced by encapsulating the optimized mRNA that encodes the RSV preF protein in self-developed lipid nanoparticles (LNPs). There are six codon mutations in the mRNA sequence to stabilize the prefusion form of the F protein and increase the preF protein expression. Here, we demonstrated that STR-V003 was able to induce RSV preF-specific IgG antibodies and neutralizing antibodies against RSV in mice and cotton rats. Furthermore, STR-V003 vaccine could significantly reduce the RSV viral loads in the lung and nose tissue of challenged animals. Meanwhile, STR-V003 did not cause any obvious enhancement of lung pathology. In addition, the repeated dose general toxicology studies and local tolerance studies of STR-V003 were evaluated in rats. Collectively, the results showed that STR-V003 had an acceptable safety profile and provided efficient protection against RSV infection without causing vaccine-enhanced disease (VED) in both mouse and cotton rat RSV infection models, suggesting that STR-V003 is a potential vaccine candidate to prevent RSV infectious diseases and has been approved by the FDA for IND.

## 2. Materials and Methods

### 2.1. Cells and Viruses

293T cells were purchased from Cell Bank/Stem Cell Bank, Chinese Academy of Sciences, HT1080 cells were purchased from Nanjing Cobioer Biosciences (Nanjing, China) and A549 cells were donated from Westlake University (Hangzhou, China). 293T, HT1080 and A549 cells were grown in complete medium (DMEM medium supplemented with 10% FBS) at 37 °C in 5% CO_2_. FI-RSV is produced by WuXi Discovery Biology (Shanghai, China). Respiratory syncytial virus, RSV/A2 (ATCC, VR-1540, Manassas, VA, USA) and RSV/B9320 (ATCC, VR-955) were used for the detection of neutralizing antibody.

### 2.2. Preparation of mRNA

In vitro transcription was performed using the TranscriptAid T7 High Yield Transcription Kit (Thermo K0441, Waltham, MA, USA), using linear DNA as a template for generating mRNA, with the addition of a certain proportion of pseudouridine and capping reagents. In vitro transcription conditions: the reaction system was configured according to the kit instructions, reaction at 37 °C for 0.5–2 h, the transcript was digested for 30 min by DNase, and the transcript was purified with Monarch RNA Cleanup Kit (NEB T2040L, Ipswich, MA, USA).

### 2.3. Lipid Synthesis

All lipids were synthesized using a similar method. General materials and solvents were purchased from Sigma Aldrich, Titan Scientific, SINOPEG and Leyan.

1-((3-((3-aminopropyl)(methyl)amino)propyl)amino)decan-2-ol: 2-octyloxirane (12.0 g, 1.0 eq.) was added to a solution of N1-(3-aminopropyl)-N1-methylpropane-1,3-diamine (44.62 g, 4.0 eq.) in EtOH (446.2 mL, 10V), then the mixture was heated to 70 °C and stirred for 15 h. Thin layer chromatography (TLC) showed the reaction was completed. The solution was evaporated under reduced pressure. H_2_O (500 mL) was then added to the residue, and the solution was washed with dichloromethane (DCM) (500 mL × 5); then, the combined organic phase was washed with saturated brine (300 mL) and dried over Na_2_SO_4_. After removing the solvent under vacuum, compound **A1-EP10** was obtained as a colorless oil (18 g, 77.74% yield). ^1^H Nuclear Magnetic Resonance (NMR) (400 MHz, CDCl3) δ 3.60 (dddd, J = 9.8, 7.2, 4.4, 2.9 Hz, 1H), 2.79–2.57 (m, 5H), 2.47–2.34 (m, 5H), 2.20 (s, 3H), 1.64 (dp, J = 18.7, 6.9 Hz, 4H), 1.49–1.18 (m, 14H), 0.93–0.81 (m, 3H).

(9Z,12Z)-octadeca-9,12-dien-1-yl acrylate: acryloyl chloride (9.76 g, 1.15 eq.) was added to a solution of (9Z,12Z)-octadeca-9,12-dien-1-ol (25.0 g, 1.0 eq.) in DCM (1250 mL, 50 V) at 0 °C, then the mixture was stirred at 0 °C for 0.5 h. A solution of Et_3_N (14.24 g, 1.5 eq.) in DCM (50 mL) was added and stirred at 0 °C for 2 h. TLC showed the reaction was completed. H_2_O (500 mL) was then added to the mixture, the organic phase was separated, and the aqueous phase was extracted by DCM (500 mL × 3); then, the combined organic phase was washed with saturated brine (300 mL) and dried over Na_2_SO_4_. After removing the solvent under vacuum, the crude product was purified by silica-gel chromatography (DCM/hexane = 1:100, *v*/*v*) to give compound **O18A** as a colorless oil (29 g, 96.44%). 1H NMR (400 MHz, CDCl_3_) δ 6.40 (dd, J = 17.3, 1.5 Hz, 1H), 6.12 (dd, J = 17.4, 10.4 Hz, 1H), 5.81 (dd, J = 10.4, 1.5 Hz, 1H), 5.49–5.26 (m, 4H), 4.15 (t, J = 6.7 Hz, 2H), 2.77 (t, J = 6.4 Hz, 2H), 2.05 (q, J = 6.8 Hz, 4H), 1.72–1.62 (m, 2H), 1.41–1.24 (m, 17H), 0.89 (t, J = 6.8 Hz, 3H).

Di((9Z,12Z)-octadeca-9,12-dien-1-yl) 3,3′-((3-((3-((2-hydroxydecyl)(3-(((9Z,12Z)-octadeca-9,12-dien-1-yl)oxy)-3-oxopropyl)amino)propyl)(methyl)amino)propyl)azanediyl)dipropionate: a solution of (9Z,12Z)-octadeca-9,12-dien-1-yl acrylate (18.6 g, 3.5 eq.) and 1-((3-((3-aminopropyl)(methyl)amino)propyl)amino)decan-2-ol (5.0 g, 1.0 eq.) was stirred at 20 °C for 1 h under N_2_, then BHT (365.41 mg, 0.1 eq.) and AcOH (49.79 mg, 0.05 eq.) was added and stirred at 70 °C for 80 h. TLC showed the reaction was completed. The mixture was purified by silica-gel chromatography (DCM/MeOH = 30:1, *v*/*v*) to give compound A1-EP10-O18A as a yellow oil (43g, yield 57.03%). ^1^H NMR (400 MHz, CDCl_3_) δ 5.45–5.24 (m, 12H), 4.11–3.98 (m, 6H), 3.63–3.53 (m, 1H), 2.95–2.84 (m, 1H), 2.81–2.53 (m, 12H), 2.49–2.15 (m, 18H), 1.67–1.53 (m, 10H), 1.51–1.15 (m, 65H), 0.88 (td, *J* = 6.8, 4.8 Hz, 12H). HR-MS: [M + H]^+^ = 1263.15105.

### 2.4. Lipid Nanoparticle Preparation and In Vivo Imaging Study

mRNA was dissolved in citrate buffer (pH 4, self-prepared from sodium citrate dihydrate and citric acid), and the concentration of mRNA adjusted to 0.2 mg/mL, thereby obtaining the aqueous phase. **A1-EP10-O18A** (synthesized according to our patented method), 1,2-distearoyl-sn-glycero-3-phosphocholine (DSPC), cholesterol and DMG-PEG2000 were dissolved in desired molar fractions in dry ethanol, and the total concentration of lipids was adjusted to 10 mg/mL, thereby obtaining the organic phase. The aqueous phase and organic phase were admixed in a 3:1 ratio (*v/v*) by a microfluidic device (NanoAssemblr^®^ Ignite™, Marlborough, MA, USA) at a total flow rate of 12 mL/min. The mixture was 10-fold diluted with PBS buffer (pH 7.4). Ethanol was separated by tangential flow filtration (Repligen^®^, TFF, Waltham, MA, USA). The solution was concentrated to 0.1 mg/mL (mRNA concentration) and filtrated by a 0.22 μm Millipore filter to obtain mRNA-containing lipid nanoparticles.

IVIS in vivo animal imaging study. Six-week-old male C57BL/6 mice weighing about 20 g, fed in the specific-pathogen-free (SPF) feeding room were selected. Two or three mice (n = 2 or 3) were randomly selected in each group, and the FLuc mRNA-containing lipid particles prepared as described above were administered via intramuscular injection (“IM”) at the dosage of 0.25 mg/kg. After 6 h, 100 μL of 30 mg/mL D-luciferin (potassium salt) was intraperitoneally injected into each mouse. After 10 min, the total fluorescence intensity of each mouse was observed and recorded by an in vivo imaging system, and the total fluorescence intensity (p/s) was recorded.

### 2.5. mRNA Transfection

293T, HT1080 and A549 cells were inoculated in 6-well or 24-well plates. Twenty-four hours later, the cells were transfected with mRNA of RSV preF protein using Lipofectamine 2000 (Invitrogen, Waltham, MA, USA). Cells were collected 24 h after transfection. The expression of RSV preF protein was then detected by Western blot with a monoclonal antibody that recognizes RSV F protein (Sinobiological, 11049-R302, Beijing, China). The expression levels of RSV F and preF proteins were analyzed by FACS in cells transfected with different mRNAs. Briefly, after transfection, cells were trypsinized and washed. F488 Anti-Respiratory Syncytial Virus Fusion Protein Antibody (Φ) (recognize the antigenic site Φ of RSV preF protein, Vazyme, DD3941-02, Nanjing, China), RSV F Antibody (4D5) (Epitope I, novoprotein, DA102), RSV F Antibody (11A9) (Epitope II, novoprotein, DA091), RSV F Antibody (4B9) (Epitope III, novoprotein, DA110), RSV F Antibody (9F4) (Epitope IV, novoprotein, DA118), RSV Pre-F Antibody (7E11) (Epitope V, novoprotein, DA119) and RSV Pre-F Antibody (3C12) (Epitope Φ, novoprotein, DA101) were used to bind specifically to RSV preF protein or F protein, respectively. Except for F488 Anti-Respiratory Syncytial Virus Fusion Protein Antibody (Φ), all other groups added Rabbit Anti-Human IgG H&L (FITC) (abcam, ab6755) as a secondary antibody. The cells were washed and analyzed by FACS. Flow cytometric data were quantitatively evaluated using FlowJo software (V10.8.1).

The expression of RSV preF protein was detected by ELISA with Respiratory Syncytial Virus pre-F Elisa Kit (Φ and IV) (Vazyme, DD3939-01). Briefly, the capture antibody (recognize the RSV preF protein-specific antigenic sites Φ) was coated on a microplate. After blocking, cell lysates were added to the wells. Serial dilutions of RSV preF-11 protein standard were added to the control columns. After incubation, the plate was washed, and the detection antibody-HRP (recognize the antigenic site IV common to RSV preF protein and postF protein) was added. After incubation, tetramethylbenzidine (TMB) was added, and plates were read in a microplate reader at 450 nm/630 nm wavelengths.

### 2.6. Animal Studies

The candidate mRNA sequences were screened based on the ability to induce RSV F or preF protein-specific IgG in BALB/c mice and Sprague Dawley (SD) rats. RSV preF mRNAs with different sequence optimization strategies were produced by in vitro transcription and encapsulated in LNP STAR0225. The LNPs (in mice, 50 µL containing 5 µg mRNA; in rats, 500 µL containing 50 µg mRNA) were used to immunize mice or SD rats (3/sex/group) via intramuscular (IM) injections on Day 0 and Day 14. The RSV preF or F-specific antibody IgG titers were analyzed 2 and 5 weeks or 3 and 5 weeks after the first dose by ELISA.

In the comparison of immunogenicity of STR-V003 and mRNA-1345, BALB/c mice (6~8 weeks old) in each group were injected with 50 µL mRNA-LNPs twice on Day 0 and Day 14 or Day 21. Vehicle was used as negative control. Serum samples were collected on Days 14, 28, and 35 for the detection of RSV preF-specific IgG antibody or neutralization antibody titers.

In the study of immunogenicity and challenge protection of STR-V003 in mouse model, female and male, 6–8 weeks old, BALB/c mice were immunized with vehicle, test vaccine STR-V003, FI-RSV by IM route on Day 0 and Day 21. Mice were deeply anesthetized by intraperitoneal injection of anesthetic (30 mg/kg Zoletil 50 and 6 mg/kg Xylazine Hydrochloride) on the day of inoculation (Day 35) and then inoculated with the RSV/A2 via intranasal inhalation. The inoculation amount is 1.0 × 10^6^ PFU/50 μL/mouse. Serum samples were collected on Days 14, 28, and Day 35 for the detection of RSV preF-specific IgG antibody and neutralization antibody titers. On the 40th day, the animals were euthanized by overdose anesthesia and exsanguination, and the lung tissue samples were collected for the detection of viral loads and lesions of lung tissue. After immunization, mice were weighed once per day for 6 consecutive days until the abnormal symptoms significantly improved and recovered, and then three times per week. After virus infection, mice were weighed once per day to observe and record their health and survival status.

In the study of immunogenicity and challenge protection of STR-V003 in cotton rat model, 5–7 week-old cotton rats were immunized with vehicle and test STR-V003 by the IM route on Day 0 and Day 21 and anesthetized with isoflurane gas in a gas anesthesia machine on the day of inoculation (Day 50). Each animal was intranasally instilled with 50 μL of viral solution, and the animals were observed to inhale the viral solution and returned to the home cage after waking up. The status of the animals was closely observed. The inoculation amount was 1.5 × 10^6^ PFU/50 μL/cotton rats. Serum samples were collected on Day 49 for neutralizing antibody. The turbinate bones and lung tissues were collected on Day 54 after the animals were euthanized for detection of viral loads and lesions of lung tissue. Animals were weighed and recorded three times per week before infection and once per day after infection.

The protocol and procedures involving the care and use of animals in the studies presented in Section 4.3, Section 4.4 and Section 4.5 were reviewed and approved by the Institutional Animal Care and Use Committee (IACUC) of GemPharmatech Co., Ltd. (Nanjing, China) before conducting the study. The studies in Section 4.6 were approved by the WuXi AppTec Co., Ltd. (Nantong, China). IACUC. During the study, the care and use of animals were conducted in accordance with the regulations of the Association for Assessment and Accreditation of Laboratory Animal Care (AAALAC). In the studies presented in Section 4.8, animal husbandry and management were compliant with the relevant SOPs of JOINN Laboratories (Suzhou, China), the Guide for the Care and Use of Laboratory Animals 8th (8th Edition, Institute of Laboratory Animal Resources, Commission on Life Sciences, National Research Council; National Academy Press; Washington, D.C., 2011), and the U.S. Department of Agriculture through the Animal Welfare Act (Public Law 99–198). JOINN Laboratories (Suzhou, China) is fully accredited by the Association for Assessment and Accreditation of Laboratory Animal Care International (AAALAC). All procedures and activities involving the use and welfare of animals were approved by the Institutional Animal Care and Use Committee (IACUC) at JOINN Laboratories (Suzhou, China).

### 2.7. Evaluation of RSV preF/F-Specific Antibody Titers

In the study of in vivo screening of RSV preF mRNA sequence for STR-V003, the titers of serum-specific IgG antibody against RSV preF of mouse were tested using an ELISA kit (Vazyme, DD3910-01) according to the manufacturer’s instructions. Briefly, the microplate was precoated with anti-pre-F IgG and preF protein. Serum dilutions were added to the wells. Positive control and negative control were added to the control columns. After incubation, the plate was washed, and the anti-mouse antibodies labeled with HRP were added. After incubation, TMB was added, and plates were read in a microplate reader at 450 nm/630 nm wavelengths.

Evaluation of RSV F-specific antibody titers was conducted as previously described [13]. ELISA plates were coated with recombinant human RSV fusion glycoprotein (total-F protein, final concentration: 0.2 μg/mL) (Sino Biological Inc. 11049-V0813, Beijing, China). The coated plates were incubated with a given serum dilution, and anti-mouse antibodies labeled with HRP (Abcam, ab6728, Cambridge, UK) were used to measure the binding of antibodies specifically to RSV-F protein using TMB substrate. Endpoint titers were defined as the reciprocal of the endpoint dilution, where the optical density (OD) signal of the serum sample was equal to or greater than twice the background OD signal.

In mouse model challenge experiments, the titers of serum-specific IgG antibody against RSV preF of mouse infection experiments were measured by ELISA. Briefly, 50 µL of the diluted antigen (HRSV (A) Pre-fusion glycoprotein F0, Acro Biosystems (RSF-V52H7, Beijing, China) at 2.0 µg/mL was added to the 96-well plate; the plate was sealed and left overnight at 2–8 °C. The plate was taken out and coated with the specific antigen from the refrigerator at 4 °C and washed once with washing solution (BIOLEGEND, 421601, San Diego, CA, USA). Then, 100 µL of blocking solution was added to each well, and the plate was sealed and incubated at 37 °C for 1.5 h. The blocking solution was discarded and washed once with washing solution. The serum samples were serially diluted with diluent. Then, 50 µL of the diluted sample or ELISA assay diluent (BIOLEGEND, 421203) was added to each well, and the plate was sealed and incubated at room temperature for 2 h. The liquid in the well was discarded and washed 3 times with washing solution. The HRP (Goat Anti-Mouse IgG H&L, Abcam, ab97023, Cambridge, UK) antibody was diluted with diluent 1:5000, 50 µL of HRP antibody added to each well; the plate was sealed and incubated at room temperature for 45 min in dark. Then, the liquid in the well was discarded and washed 3 times with washing solution. Chromogenic reagent A solution and B solution (BIOLEGEND, 421101) were mixed at 1:1, and 50 µL chromogenic solution was added to each well and incubated in the dark for 10 min. Then, 50 µL of stop solution (BIOLEGEND, 423001) was added to each well. The absorbance values at 450 nm were measured using a multi-functional microplate reader, the OD450 values were output, and the RSV-preF IgG antibody titers of serum samples of each animal were analyzed using a four-parameter fitting method.

### 2.8. Neutralizing Antibody

RSV neutralizing antibody was measured to evaluate the immunogenicity of STR-V003. Neutralizing antibody was detected by microneutralization. Briefly, the heat-inactivated serum samples were first diluted 20 times with DMEM, and then a 3-fold serial dilution was made; a total of 8 points were diluted. Serial dilutions of heat-inactivated serum from vaccinated or control animals were incubated with virus (200 TCID50/well) for 2 h at 37 °C. The serum–virus mixture or virus alone was added to the wells containing Hep-2 cells (ATCC, Manassas) and incubated for 2 h or 5 days at 37 °C. After removing the supernatant, a culture medium was added, and the culture continued for about 22 h. The cells were then fixed, and virus-specific foci were detected by antibody against RSV F protein (Sino Biological, 11049-R338, Beijing, China) or RSV G protein (Vazyme, DD1606, Nanjing, China), and secondary antibody (HRP-labeled Goat Anti-Rabbit IgG (H+L), Beyotime Institute of Biotechnology, A0208, Shanghai, China). The signal was developed by TMB, and absorbance was read at 450 nm. NT50 (the reciprocal of the serum dilution ratio when RSV viruses suppressed by 50%) was calculated to indicate the neutralizing activity of antibody against RSV viruses.

### 2.9. Quantification of Viral RNA in the Lung Tissue or Turbinate Bones of the Infected Animal by RT-qPCR

To evaluate the efficacy of the vaccine in the mouse and cotton rat model challenge experiment, RSV viral loads in lung tissue and turbinate bone were measured by RT-qPCR. The tissue samples were divided into several small pieces, and 600 μL RLT lysate (RNeasy Mini Kit, QIAGEN, 74104, Shanghai, China) was added to grind the lung tissue in a tissue homogenizer. Then, 100 μL of the lysate was used for RNA extraction and eluted with 50 μL of nuclease-free water (RNeasy Mini Kit) to obtain the total RNA. For turbinate bone, 150 μL of the lysate was added to 850 μL QIAzol (QIAGEN, 79306, Shanghai, China) for RNA extraction and eluted with 50 μL of nuclease-free water (RNeasy Mini Kit) to obtain total RNA. RSV-specific reverse transcription primers (GENEWIZ, Suzhou, China) were used with 10 μL RNA to synthesize cDNA. qPCR primers, probes (primers and probes were synthesized by GENEWIZ, Suzhou), FastStart Universal Probe Master (ROX) (Sigma-Aldrich, 04914058001, St. Louis, MO, USA), and water were mixed to prepare a qPCR reaction system, and 1 μL of cDNA or diluted plasmid RSV-N (GENEWIZ, Suzhou) was added as a template for TaqMan qPCR for detection. Using plasmid RSV-A-N or RSV-B-N copy number and qPCR amplification cycle number, a standard curve was created, the RSV copy number of all tested samples was calculated, and finally, the RSV virus copy number per gram of tissue was calculated.

### 2.10. Histopathology Assay

Histopathology assays were conducted as previously described [13]. After perfusion, the lung tissue was fixed in 4% paraformaldehyde for more than 24 h. The next step was carried out in the pathological laboratory, where the tissue was dehydrated, followed by embedding and slicing with a thickness of 4 μm. The staining rack loaded with slices was placed in an oven at 60–65 °C for 1 h. After the paraffin melted to a transparent liquid state, the staining rack was placed at room temperature for 10 min to cool. After cooling, slices were placed in hematoxylin and eosin (HE) staining and sealed. Finally, lung sections were scored by a person blinded to the group assignment, and the scoring criteria are shown in Supplementary Table S3.

### 2.11. Safety Study of STR-V003

A total of 140 Sprague Dawley (SD) rats (70 animals/sex) were randomly assigned to 14 groups, with 5 animals/sex/group in Groups 1 to 7 designated as the main study groups and 5 animals/sex/group in Groups 8 to 14 as the satellite groups (Supplementary Table S5). Rats were treated with 0.9% sodium chloride injection (Hunan Kelun Pharmaceutical Co., Ltd., Changsha, China) (1 mL/animal) as negative control for Group 1 and 8, STR-V003 (mRNA content of 50 μg/dose, 100 μg/mL, 0.5 mL/dose) at doses of 0.5 mL/1, 0.75 mL/1.5 and 1 mL/2 dose/animal for animals of Groups 2 and 9, Groups 3 and 10 or Groups 4 and 11, respectively, empty LNPs at doses of 0.5 mL/1, 0.75 mL/1.5 and 1 mL/2 dose/animal for animals of Groups 5 and 12, Groups 6 and 13 or Groups 7 and 14, respectively. The animals were administered treatment via intramuscular injection once every two weeks for 4 consecutive weeks (3 doses in total).

Parameters evaluated in this study included clinical observations (including administration site observations), body weight, food consumption, body temperature and hematology. The animals in Groups 1 to 7 and Groups 8 to 14 were euthanized 3 days after the last dosing (Day 32) and after the end of the recovery period (Day 43) for macroscopic changes, organ weight and histopathological examination of the liver, submandibular lymph nodes, mesenteric lymph nodes, inguinal lymph nodes, spleen and thymus.

For the non-human primate (NHP) study, there were middle-aged female cynomolgus monkeys, among which 3 were in the vehicle group and 5 were dosed with STR-V003 analogue. The mRNA vaccine was injected intramuscularly on Day 0 and Day 21, which was consistent with our clinical plan. Blood was collected within 24 h after each administration to detect the blood biochemical and cytokine secretion conditions. The whole NHP experiment lasted for approximately 2 months, and the changes in body weight and body temperature were monitored and completed in the Saifu Laboratories Co., Ltd. (Beijing, China).

## 3. Statistical Analysis

GraphPad Prism 7.00 was used for statistical analysis of the data. One-way ANOVA was used to analyze the statistical difference of lung tissue viral loads. One-way ANOVA and two-way ANOVA were used to analyze the statistical difference in lung tissue pathology.

For the safety study of STR-V003/Empty LNPs, all statistical tests were conducted as 2-sided tests, and the level of significance was set at 5% or *p* ≤ 0.05. Group means and standard deviation (SD) were calculated for males and females, respectively, in the Provantis system (SAS 9.4 software), including body weights, body weight gain, organ weights, organ-to-body/brain weight ratios, clinical pathology (hematology), etc. in the negative control article and dose groups. The data were analyzed with the following procedures:

Levene’s test was performed on the original data to test for variance homogeneity. When the result showed no significance (*p* > 0.05), a one-way analysis of variance (ANOVA) was performed. If ANOVA showed significance (*p* ≤ 0.05), Dunnett’s test (parametric method) was performed for multiple comparisons.

In the case of variance heterogeneity (*p* ≤ 0.05 by Levene’s test), the data were logarithmically transformed using the natural logarithm (ln transformation), and then the transformed data were tested for variance homogeneity as described above using Levene’s test. In the case of variance heterogeneity of transformed data (*p* ≤ 0.05 by Levene’s test) or negative values, a Kruskal–Wallis test (non-parametric method) was performed on the original data. If the Kruskal–Wallis test showed significance (*p* ≤ 0.05), a two-independent-sample test (Mann–Whitney U Wilcoxon) was performed for multiple comparisons.

When a dataset has zero values, the zero values were regarded as 1/10 of the smallest positive value in the dataset when logarithmical transformation is performed.

## 4. Results

### 4.1. Lipid Synthesis and Screening of LNP Formulations

**Figure 1:**
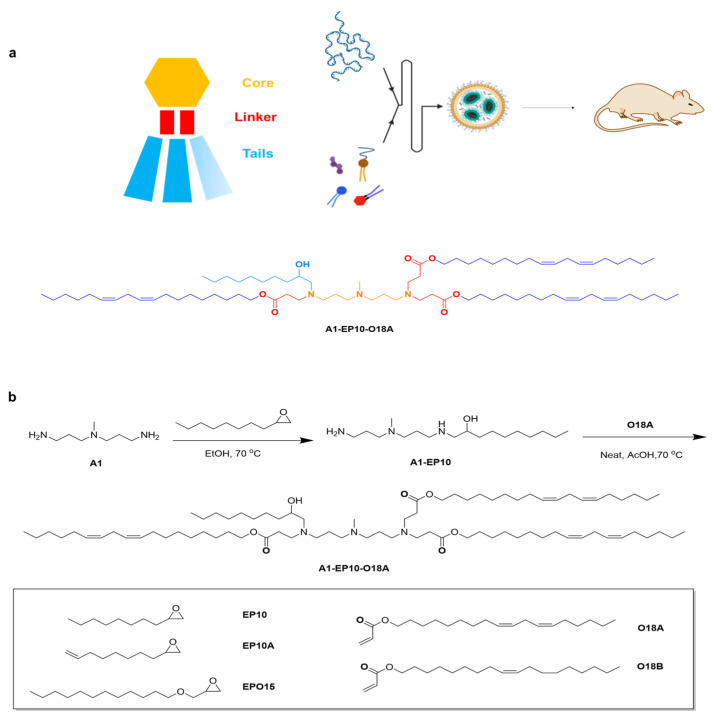

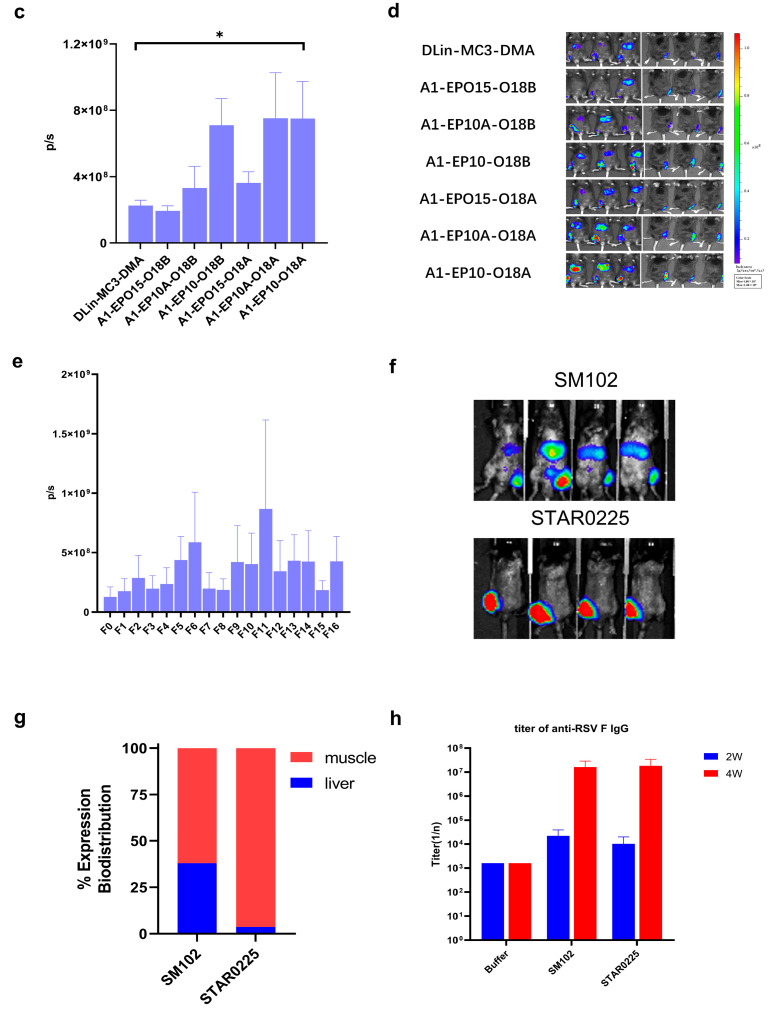
Design, screening and optimization of LNP formulation. (**a**) Schematic of AAiL structure and LNP formulating for mRNA delivery. (**b**) Structure of tails and synthetic route toward A1-EP10-O18A. (**c**) In vivo delivery efficacy of luciferase mRNA in C57BL/6 mice (0.25 mg/kg mRNA per mouse, through I.M. injection, n = 3, mean ± SEM). Luminescence was recorded 5 h after injection. (**d**) Imaging of body was recorded 5 h after injection. (**e**) DOE study results of in vivo delivery efficacy of luciferase mRNA in C57BL/6 mice (0.25 mg/kg mRNA per mouse, through I.M. injection, n = 2, mean ± SEM). Luminescence recorded 5 h after injection. (**f**,**g**) Comparison of luminescence of STAR0225 and SM102 LNP in C57BL/6 mice (0.25 mg/kg through I.M. injection). (**h**) Comparison of wild-type RSV F mRNA encapsulated with STAR0225 and SM102 LNP in BALB/c mice (5 μg/mice through I.M. injection).

The asymmetric alkanolamine ionizable lipid (AAiL) library was composed of three structural components: an amine core, a biodegradable linker, and a tail linker. The AAiL was crafted through a precise Michael addition process between amine alcohols and acrylates through rational design (Figure 1a,b). These AAiL synthetic products were then formulated with DSPC, cholesterol, DMG-PEG2000, and luciferase (Luc) mRNA to create LNPs for an initial assessment with a fixed molar ratio of (50:10:38.5:1.5) for initial screening (Figure 1c,d and Supplementary Table S1). According to the efficacy and material accessibility, the lipid of A1-EP10-O18A was identified for further optimization using a Design of Experiment (DOE) strategy (Figure 1e, Supplementary Figures S1 and S2). The refined F11 formulation (STAR0225, Supplementary Table S2) packing with luciferase mRNA was chosen to compare with Moderna’s formulation (SM102) and showed a better local distribution. (Figure 1f,g). Consequently, we hypothesized that STAR0225 should be a promising candidate for the development of RSV mRNA vaccines.

We conducted a straightforward comparative experiment to quickly validate this hypothesis by investigating the immunogenicity of mRNA, coding wild-type (WT) F protein of the RSV A2 strain encapsulated in SM102 and STAR0225 in mice. The RSV F-specific antibody IgG titers were analyzed 2 and 4 weeks after the first dose by ELISA. As shown in Figure 1h, WT-SM102 and WT-STAR0225 induced comparable RSV F-specific antibody IgG titers, supporting the further development of an RSV mRNA vaccine with STAR0225.

### 4.2. In Vitro Screening of RSV preF mRNA Sequence for mRNA Vaccine

**Figure 2:**
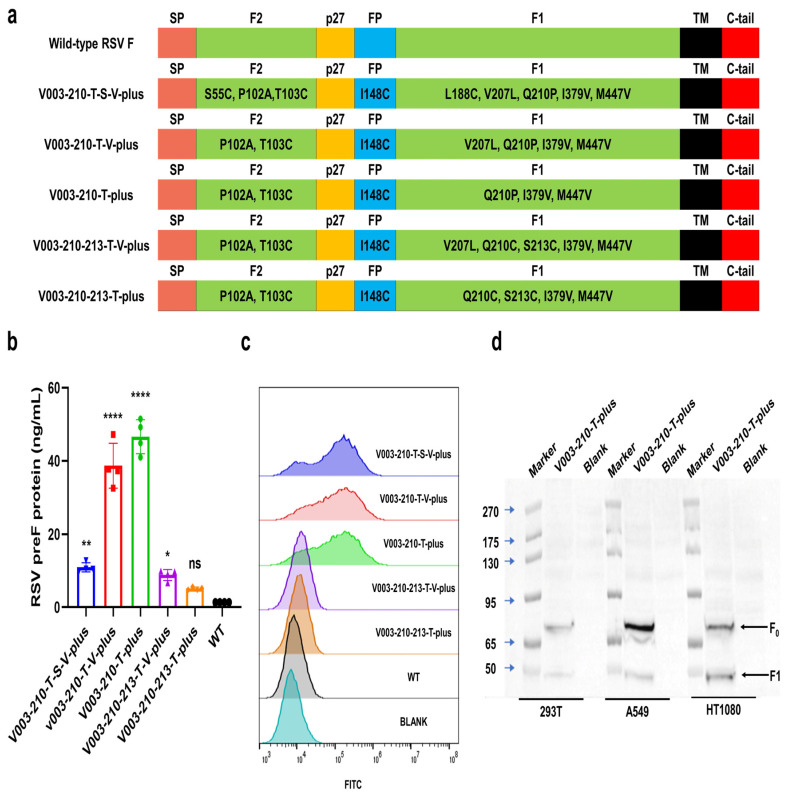
Sequence screening of RSV F protein mutants expressed from mRNA constructs in vitro. (**a**) RSV F proteins encoded by different mRNA constructs. Wild-type (WT) RSV F encoding the F protein of the prototype RSV A2 strain. SP, signal peptide; F2, a polypeptide of F protein; p27, p27 peptide; FP, fusion peptide; F1, a polypeptide of F protein; TM, transmembrane peptide; C-tail, cytoplasmic tail; point mutations are labeled. (**b**) Expression level of RSV preF proteins in 293T cells. (**c**) Expression of RSV preF proteins in HT1080 cells detected by FACS. Fluorescent (Alexa Fluor 488)-labeled antibody recognizing antigenic site Φ (specific for RSV preF protein) was used to stain RSV preF protein. (**d**) Maturation of RSV F protein. F_0_, inactive precursor of F protein. Data are shown as mean ± SEM, and asterisks indicate significant (*: *p* < 0.05, **: *p* < 0.01, ****: *p* < 0.0001).

The majority of highly neutralizing antibodies induced by natural infection or immunization targets the RSV preF form, making it the preferred antigen for vaccine development. In order to improve the efficiency of RSV mRNA vaccine, following strategies used formerly for SARS-CoV-2 S protein and RSV F protein, such as engineered disulfide mutations, electrostatic mutations, cavity filling mutations and trimerization domain [5,14,15,16,17], we constructed F protein mutants to increase expression yields of F and preF protein by the introduction of the amino acid mutations, including amino acid substitutions, deletions, or additions relative to a wild-type F protein in our earlier studies (PCT/CN2024/083234). Combined with the findings in the in vitro expression study, we finally selected five mutant mRNAs for subsequent studies (Figure 2a).

To optimize the mRNA sequence used in the vaccine, five mRNA constructs encoding RSV F and preF proteins were transfected into HT1080, A549, and 293T cells. As illustrated in Figure 2b,c, ELISA and FACS analyses for detecting the expression levels of RSV preF proteins in cells revealed that compared with the mRNA of WT F protein, all mutant mRNAs resulted in higher expression levels of preF proteins 24 h post-transfection. Taken together, V003-210-T-plus is among the mRNA constructs that generally yielded the highest expression under different experiment conditions. Moreover, RSV F protein-specific antigenic sites of V003-210-T-plus were detected by FACS (Supplementary Figure S3). Except for antigenic site I, all other antigenic sites could be identified. It might be possible for antibody against antigenic site I binding to be tighter to the post-F conformation than pre-F conformation.

The inactive precursor, RSV F_0_, requires cleavage during intracellular maturation by a furin-like protease [18,19]. RSV F contains two furin sites, which leads to three polypeptides: F2, p27(a 27-amino acid fragment) and F1 [20,21,22]. The cleavage process was analyzed by Western blot in HT1080, A549 or 293T cells transfected with mRNA V003-210-T-plus. As shown in Figure 2d, two forms of RSV F proteins were detected, which migrate at ~65kDa (RSV F_0_) and ~50 kDa (RSV F1). The detection of the faster-migrating variant of RSV F protein confirmed the cleavage of inactive precursor (RSV F_0_).

### 4.3. In Vivo Screening of RSV preF mRNA Sequence In Mice and Rats

**Figure 3:**
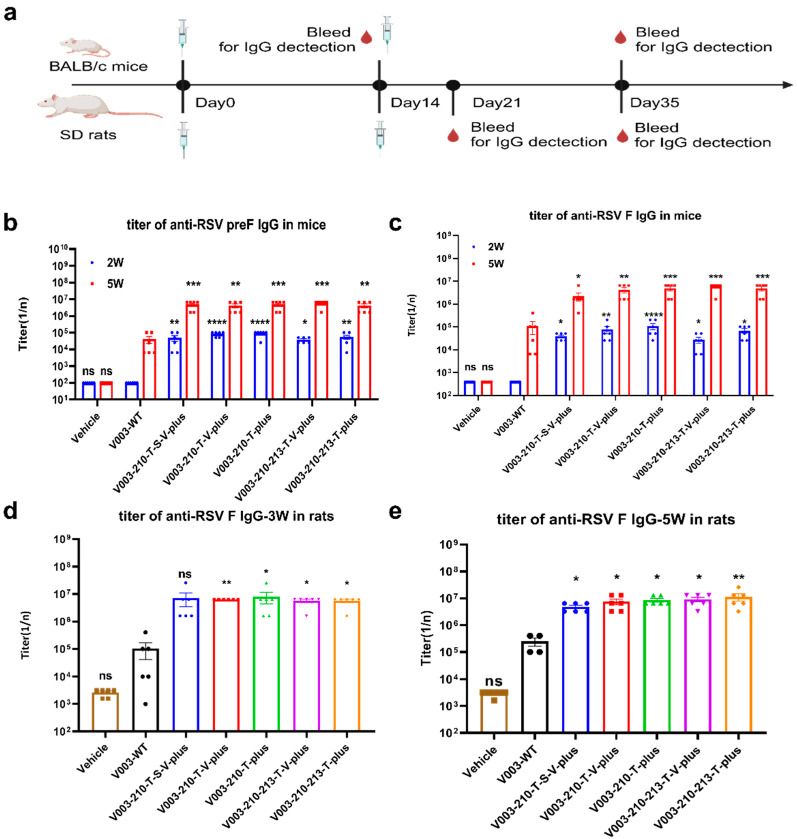
Sequence screening of RSV F protein mutants expressed from mRNA constructs in vivo. (**a**) The schematic diagram of the immunogenicity of STR-V003 in mouse and rat models (created with BioRender.com). RSV F mRNAs with different sequences encapsulated in LNPs. The LNPs (in BALB/c mice, at 50 µL containing 5 µg mRNA, in SD rats, at 500 µL containing 50 µg mRNA) were used to immunize mice (3/sex/group) and SD rats (3/sex/group) via IM injections on Day 0 and 2 weeks. The RSV preF/F-specific antibody IgG titers were analyzed 2 and 5 weeks after the first dose by ELISA. (**b**,**c**) Titers of RSV preF/F-specific antibodies in mice at 2 and 5 weeks. (**d**,**e**) Titers of RSV F-specific antibodies in rats at 2 and 5 weeks. Endpoint titers were defined as the reciprocal of the endpoint dilution, where the optical density (OD) signal of the serum sample was equal to or greater than twice the background OD signal. Data are shown as mean ± SEM, and asterisks indicate significant differences (*: *p* < 0.05, **: *p* < 0.01, ***: *p* < 0.001, ****: *p* < 0.0001, compared with V003-WT at 2 W or 5 W).

The candidate mRNA sequences were screened based on the ability to induce RSV F or preF protein-specific IgG in BALB/c mice and Sprague Dawley (SD) rats (Figure 3a). As shown in Figure 3b,c, compared with the V003-WT, all mutant mRNAs induced higher RSV F and RSV preF-specific antibody IgG titers in mice at both 2 weeks and 5 weeks after the first dose. Compared with the first dose in mice, the second dose of vaccine led to higher titers of RSV F and RSV preF-specific antibody. As shown in Figure 3d,e, compared with the V003-WT, all mutant mRNAs induced higher RSV F-specific antibody IgG titers in rats at both 3 weeks and 5 weeks after the first dose. Compared with the first dose in rats, the second dose of higher vaccine-induced RSV F-specific antibody IgG titers demonstrated that the second dose of vaccine further boosted the immune response.

Taken together, V003-210-T-plus generally induced the highest RSV preF-specific antibody titers and RSV F-specific antibody titers. Combined with the findings in the in vitro expression study, these results suggested that V003-210-T-plus is the optimal mRNA sequence that can be used in the STR-V003 vaccine.

### 4.4. Comparison of Immunogenicity of STR-V003 and mRNA-1345 Analog in Mice

**Figure 4:**
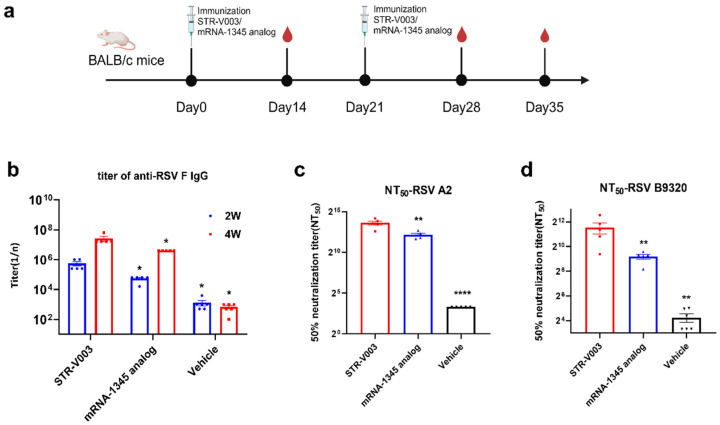
Comparison of immunogenicity of STR-V003 and mRNA-1345 analog in mice. (**a**) The schematic diagram of the immunogenicity of STR-V003 and mRNA-1345 analog in mouse model (created with BioRender.com). STR-V003 and mRNA-1345 analog (both at 5 μg) were used to immunize (IM injections at Day 0 and Day 21) mice (5/sex/group). Serum samples were collected on Days 14, 28 and 35. (**b**) RSV F-specific IgG antibody in mouse serum on Days 14 and 28. (**c**,**d**) The 50% neutralizing titer (NT_50_) against RSV/A2 and RSV/B9320 in mice on Day 35. Data are shown as mean ± SEM, and asterisks indicate significant differences (*: *p* < 0.05, **: *p* < 0.01, ****: *p* < 0.0001).

Moderna’s mRNA-based mResvia, mRNA-1345, was the third RSV vaccine approved by the FDA. We compared the immunogenicity of STR-V003 and mRNA-1345 analog in mice (Figure 4). Here, mRNA-1345 analog and mRNA-1345 had the same LNP formulation and CDS sequences of mRNA (WO2021/155243 A1). STR-V003 and mRNA-1345 analog (both at 5 μg) were used to immunize (IM injections at Day 0 and Day 21) mice (5/female/group). Serum samples were collected on Days 14, 28 and 35. The RSV F-specific antibody IgG titers were analyzed 2 and 4 weeks after the first dose by ELISA. The titer of neutralizing antibodies in serum samples of 5 weeks was detected by microneutralization. As shown in Figure 4b–d, STR-V003 induced higher levels of RSV F-specific antibody IgG titers and neutralizing antibody titers than mRNA-1345 analog.

### 4.5. Optimization of Administration Route, Dose-Response Assessmtion Regimen in BALB/c Mice

**Figure 5:**
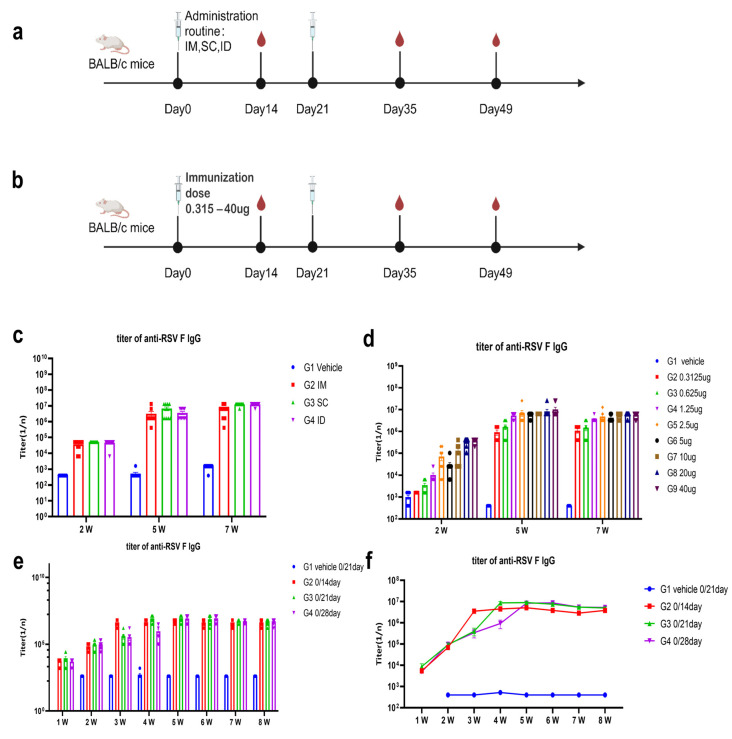
Optimization of administration route, dose response and immunization regimen in BALB/c mice. (**a**) Optimization of administration route in BALB/c mice (created with BioRender.com). V003-210-T-plus mRNA was encapsulated in LNPs. The LNPs (50 µL, containing 5 µg mRNA) were used to immunize mice (5/sex/group) via different routes on Day 0 and Day 21. The immunogenicity was evaluated 2, 5 and 7 weeks after the first dose by measuring RSV F-specific antibody (IgG) titers. IM, intramuscular; SC, subcutaneous; ID, intradermal. (**b**) Optimization of dose–response in BALB/c mice (created with BioRender.com). The LNPs (approximately 50 µL, containing 0.3125, 0.625, 1.25, 2.5, 5, 10, 20, or 40 µg V003-210-T-plus mRNA) were used to immunize mice (5/sex/group) via IM injections on Day 0 and Day 21. Titers of RSV F-specific antibodies in mice at 2, 5 and 7 weeks were evaluated. (**c**) Titers of RSV F-specific antibodies in mice at 2, 5 and 7 weeks via different administration routes. (**d**) Titers of RSV F-specific antibodies in mice at 2, 5 and 7 weeks with different immunization doses. (**e**,**f**) The LNPs (50 µL containing 5 µg V003-210-T-plus mRNA) were used to immunize mice (5/sex/group) via IM injections following different dosing regimens (Day 0 and Day 14, Day 0 and Day 21, Day 0 and Day 28). The immunogenicity was evaluated once weekly for up to 8 weeks after the first dose by measuring RSV F-specific antibody (IgG) titers. Data are shown as mean ± SEM.

To optimize the route of immunization, V003-210-T-plus encapsulated in LNPs (STR-V003) were used to immunize mice (5/sex/group) via different routes (IM, subcutaneous (SC) or intradermal (ID) injections) on Day 0 and Day 21. Compared to the vehicle IM group, STR-V003 administered via different routes (IM, SC, and ID) all yielded similarly higher immunogenicity in mice (Figure 5a,c). The most common clinical vaccination route, IM, was selected for further studies [23].

To understand the effects of immunization doses on the induced immunogenicity, different doses of STR-V003 were used to immunize mice (5/sex/group) via IM injections on Day 0 and Day 21 (Figure 5b). At 2 weeks, RSV F-specific antibody IgG titers increased in a dose-dependent manner when the dose increased from 0.3125 to 40 μg. As shown in Figure 5d, compared with those at 2 weeks, RSV F-specific antibody IgG titers increased at 5 weeks, suggesting that the second dose of vaccine further boosted the immune response. RSV F-specific antibody IgG titers were not significantly different between 7 weeks and 5 weeks, suggesting that immune responses to the vaccine were maintained for at least 4 weeks after the second vaccination. At both 5 and 7 weeks, RSV F-specific antibody IgG titers increased when the dose increased from 0.3125 to 1.25 μg but did not further increase when doses were higher than 1.25 μg.

To evaluate the impact of the immunization regimen on the immunogenicity elicited, STR-V003 was used to immunize mice (5/sex/group) via IM injections following different dosing regimens (Day 0 and Day 14, Day 0 and Day 21, Day 0 and Day 28).

As shown in Figure 5e,f, the 0/21-day regimen generated more potent immunogenicity than the 0/14-day regimen. Due to the earlier boost (3 weeks after the first dosing instead of 4 weeks), the 0/21-day regimen induced stronger immunogenicity than the 0/28-day regimen at 4 weeks after the first dosing. At other time points, the 0/21-day regimen induced comparable immunogenicity as the 0/28-day regimen. Taken together, the 0/21-day regimen is chosen for future trials.

### 4.6. Immunogenicity and Challenge Protection of STR-V003 in Mouse Model

**Figure 6:**
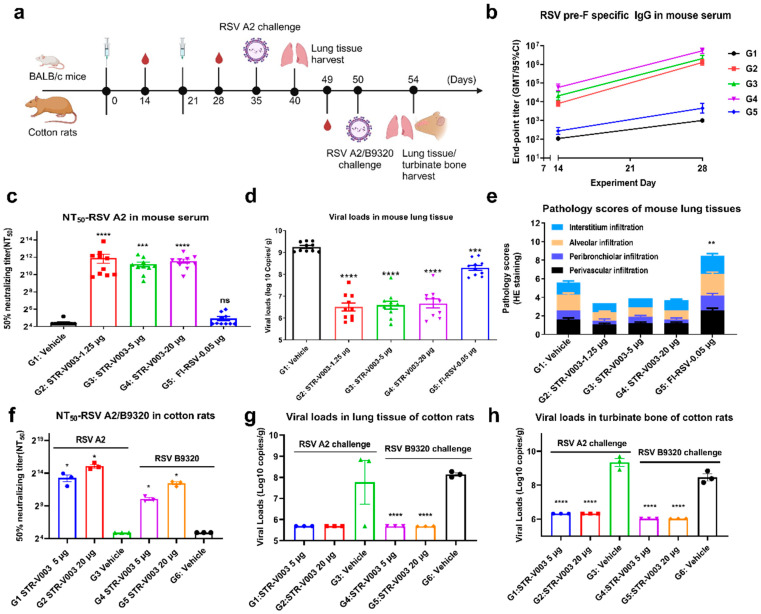
Protection agalenge protection of STR-V003 in mouse model and cotton rat model (created with BioRender.com). The formalin-inactivated vaccine (FI-RSV at 0.05 μg), STR-V003 (1.25, 5, or 20 μg) was used to immunize (IM injections at Day 0 and Day 21) mice (5/sex/group). RSV/A2 was inoculated intranasally (1.0 × 10^6^ PFU/animal) on Day 35. Serum samples were collected on Days 14, 28 and 35, and lung tissue samples were collected on Day 40. Cotton rats were immunized with STR-V003 vaccine (5 or 20 μg) via IM injections on Day 0 and Day 21. On Day 30, RSV/A2 or RSV/B9320 were inoculated intranasally (1.5 × 10^6^ PFU/animal). Serum samples were collected on Day 49. The turbinate bones and lung tissues were collected on Day 54 after the animals were euthanized. (**b**) RSV preF-specific IgG antibody in mouse serum on Days 14 and 28. (**c**) The 50% neutralizing titer (NT_50_) in mice on Day 35. (**d**) Viral loads in mouse lung tissue on Day 40. (**e**) Pathology scores of mouse lung tissues on Day 40. (**f**) The 50% neutralizing titer (NT50) of RSV A2 and RSV B9320 in cotton rats on Day 49. (**g**,**h**) Viral loads in lung tissue and turbinate bone of cotton rats on Day 54. Data are shown as mean ± SEM, and asterisks indicate significant differences (*: *p* < 0.05, **: *p* < 0.01, ***: *p* < 0.001, ****: *p* < 0.0001).

Mice and cotton rats, two commonly used models of human RSV infection, have provided insights into the pathogenesis of RSV infections and mechanisms of immunity [24,25]. The immunogenicity and challenge protection of STR-V003 were first evaluated in the mouse model. The formalin-inactivated vaccine (FI-RSV at 0.05 μg), STR-V003 (at 1.25, 5, or 20 μg) and vehicle were used to immunize (IM injections at Day 0 and Day 21) mice (5/sex/group). RSV/A2 (ATCC, VR-1540) was inoculated intranasally (1.0 × 10^6^ PFU/animal) on Day 35 (Figure 6a). Serum samples were collected on Days 14, 28 and 35, and lung tissue samples were collected on Day 40. The titer of neutralizing antibodies in serum samples was detected by microneutralization, the titer of RSV preF-specific IgG antibody in animal serum samples was detected by ELISA, the titer of RSV virus in lung tissue was detected by qPCR, and the H&E staining of lung tissue sections was evaluated for peribronchiolitis (PB), perivasculitis (PV), interstitial pneumonia (IP) and alveolitis (A), and evaluate the immunogenicity and in vivo antiviral efficacy of the tested vaccine [5]. Histopathology semiquantitative scoring criteria of lung tissue sections are shown in Supplementary Table S3.

During the study, no significant weight loss was observed. Compared with the vehicle, FI-RSV significantly reduced the RSV virus titer in the lung tissue by 0.96 Log10 (copies/g lung) (Figure 6d). The total lung pathology score in the FI-RSV group was 8.5 ± 0.79, while that in the vehicle group was 5.6 ± 0.40, indicating that FI-RSV significantly enhanced lung pathological damage (Figure 6e). This result demonstrated that while FI-RSV could provide protection against RSV infection, it elicited significant vaccine-related disease, consistent with previous reports [26,27].

Compared with the vehicle group and FI-RSV group, STR-V003 induced dose-dependent RSV preF-specific IgG antibodies (Figure 6b) and significantly higher levels of neutralizing antibodies at all dose levels (Figure 6c). Compared with the vehicle group, STR-V003 significantly reduced the RSV viral loads by 2.75, 2.67 and 2.58 Log10 (Copies/g lung) in the lung tissue of animals treated with 1.25, 5 or 20 μg vaccine, respectively (Figure 6d). As shown in Figure 6e, test vaccine STR-V003 (1.25 µg/mouse) significantly reduced perivasculitis, peribronchiolitis and alveolitis inflammation scores and a medium dose (5 µg/mouse) significantly reduced alveolitis inflammation. The high dose (20 µg/mouse) significantly reduced peribronchial and alveolitis inflammation scores. The experimental results showed that the tested vaccine had no enhancement of lung damage. Taken together, these results demonstrated that STR-V003 provided efficient protection against RSV infection without causing VED.

### 4.7. Immunogenicity and Challenge Protection of STR-V003 in Cotton Rat Model

The immunogenicity and challenge protection of STR-V003 were further evaluated in the cotton rat model. Cotton rats were immunized with STR-V003 vaccine (containing 5 or 20 μg mRNA) and vehicle via IM injections on Day 0 and Day 21. On Day 50, RSV/A2 (ATCC, VR-1540) or RSV/B9320 (ATCC, VR-955) were inoculated intranasally (1.5 × 10^6^ PFU/animal) (Supplementary Table S4). Serum samples were collected on Day 49 for neutralizing antibody. The turbinate bones and lung tissues were collected on Day 54 after the animals were euthanized (Figure 6a).

Neutralizing antibody was measured by microneutralization to evaluate the immunogenicity of STR-V003. To evaluate the efficacy of the vaccine in vivo, RSV viral loads in lung tissues and turbinate bones were measured by RT-qPCR. Compared with the vehicle, the tested vaccine STR-V003 induced dose-dependent levels of neutralizing antibodies (against RSV/A2 or RSV/B9320) (Figure 6f), significantly reduced the RSV viral loads in the lung tissue (by 2.081 Log10 (copies/g lung) for RSV A2, by 2.462 Log10 (copies/g lung) for RSV B9320 at both doses) and turbinate bone (by 3.023 Log10 (copies/g bone) for RSV A2, by 2.433 Log10 (copies/g bone) for RSV B9320 at both doses) of infected animals (Figure 6g,h). The experimental results showed that test vaccine STR-V003 had good anti-RSV virus effects in vivo.

Evaluation of lung tissue lesions by HE staining, perivascular, peribronchial, alveolar and pulmonary interstitial inflammation score results are shown in Supplementary Table S5. Compared with the vehicle group (G3), test vaccine STR-V003 group (G1–2) had no enhancement of lung damage. Compared with the vehicle group (G6), test vaccine STR-V003 group (G3–4) had no enhancement of lung damage. The experimental results showed that test vaccine STR-V003 had no enhancement of lung damage, demonstrating efficient protection against RSV infection was provided by STR-V003 without causing VED.

### 4.8. Safety of STR-V003 in Rodents and NHP

**Figure 7:**
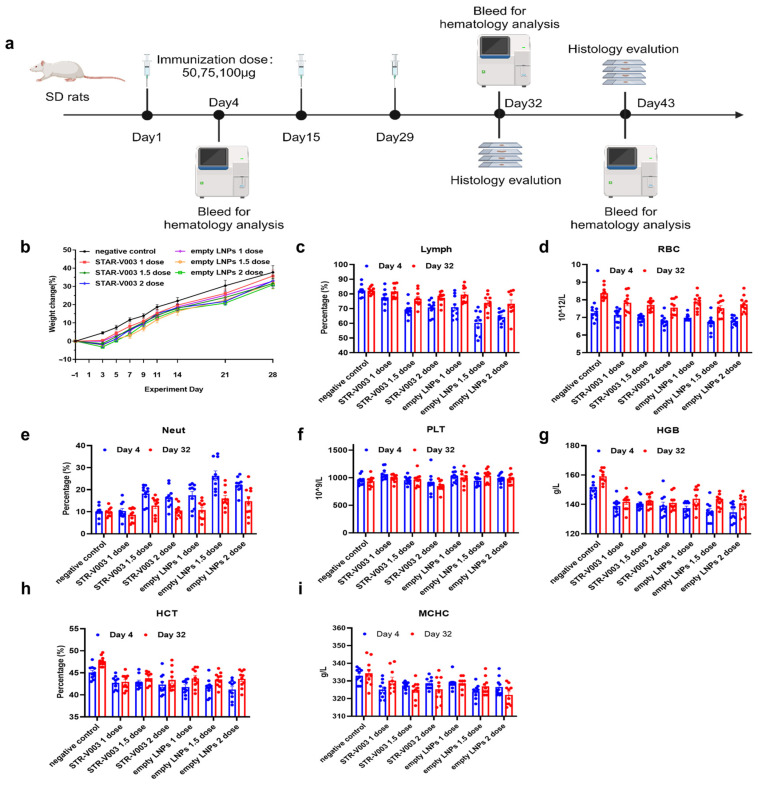
Safety of STR-V003 in SD rat. (**a**) The schematic diagram of safety studies on STR-V003 and A1-EP10-O18A in SD rats (created with BioRender.com). The SD rats received negative control (sodium chloride injection), STR-V003 at 1, 1.5 or 2 doses (50, 75 or 100 µg) or empty LNPs at 0.5, 0.75 or 1 mL (equivalent to 1, 1.5 or 2 vaccine doses) via IM injections (Q2W for a total of 3 administrations) following a 2-week recovery period. (**b**) Body weight changes of rats after immunization. (**c**–**i**) Pathological indicators in the blood of rats on Day 4 and Day 32, including lymphocyte (Lymph) percentage (**c**), erythrocyte count (RBC) (**d**), neutrophilic granulocyte (Neut) percentage (**e**), platelet (PLT) counts (**f**), hemoglobin (HGB) concentration (**g**), hematocrit (HCT) percentage (**h**), and mean corpuscular hemoglobin concentration (MCHC) (**i**). Data are shown as mean ± SEM.

Safety studies on STR-V003 and A1-EP10-O18A (as empty LNPs) were designed in accordance with the WHO guidelines on vaccines [28]. As shown in Figure 7a, the potential sub-chronic toxic effects and the reversibility, persistence or any delayed occurrence of observed adverse events were evaluated for STR-V003 and empty LNPs in a 4-week repeat-dose toxicology study with a 2-week recovery phase in SD rats. The rats were randomly assigned into seven groups (10/sex/group) and received negative control (sodium chloride injection), STR-V003 at 1, 1.5 or 2 doses (50, 75 or 100 µg), or empty LNPs at 0.5, 0.75 or 1 mL (equivalent to 1, 1.5 or 2 vaccine doses), via IM injections (Q2W, for a total of 3 administrations). On Day 32, half of the animals (5/sex/group) were euthanized and necropsied, while the remaining animals (5/sex/group) were euthanized on Day 43. Blood samples for hematology determinations were collected on Day 4, Day 32 and Day 43.

All doses of STR-V003 and empty LNPs were tolerated. There was no treatment-related mortality or moribundity. Local irritation reactions were observed at the injection sites, with a trend of recovery after a 2-week recovery period.

All doses of STR-V003 and empty LNPs were tolerated. There was no treatment-related mortality or moribundity. No empty LNPs/STR-V003-related changes in ophthalmoscopic examinations throughout the study. Swelling in the injection sites was noted in animals in all empty LNP groups and the STR-V003 groups 2 to 4 days after dosing, which recovered afterwards.

Compared with the negative control group of the same gender in the same period, decreased body weight and body weight gain were observed 1 week after each dosing in the STR-V003 and empty LNP groups (Figure 7b). A trend toward recovery was seen at Week 2 after dosing. These lower body weights were considered secondary changes to local irritancy of the dose. At the same dose, the change ranges were generally similar in the empty LNPs and the STR-V003 groups.

Compared with the negative control group of the same gender in the same period, increased Neut and decreased RBC, HGB and HCT in rats were observed on Day 4 and Day 32 in the STR-V003 and empty LNPs groups at 1, 1.5 and 2 dose/animal (Figure 7c–i). These changes were considered possibly related to an immune response and/or local irritation and/or the acute phase response [29]. A trend towards recovery or recovery was seen after 2 weeks of drug withdrawal (Day 43). At the same dose, the change ranges were similar in the empty LNP and STR-V003 groups.

The animals were euthanized at scheduled necropsy for macroscopic changes, organ weight and histopathological examination of liver, submandibular lymph nodes, mesenteric lymph nodes, inguinal lymph nodes, spleen and thymus. At 3 days after the last dosing (Day 32), thymus weight and thymus-to-body/brain weight ratios decreased, while spleen weight and spleen-to-body/brain weight ratios increased in the STR-V003 and empty LNPs groups (Supplementary Table S6). As shown in Table 1, microscopically, minimal to moderate cellularity lymphocytes of the Supplementary Table S6 tyms decreased and minimal to moderate vacuolation of hepatocytes around portal areas in the liver were observed in the STR-V003 and empty LNP groups; minimal to slight cellularity lymphocytes in the white pulp in the spleen increased in the STR-V003 and empty LNP groups at 1.5 and 2 dose/animal. Extramedullary hematopoiesis of the red pulp in the spleen increased in the STR-V003 groups at 1, 1.5 and 2 dose/animal and empty LNP groups at 1.5 and 2 dose/animal. A trend towards recovery or recovery was seen after 2 weeks of drug withdrawal (Day 43). At the same dose, the change range, incidence and severity of lesions were similar in the empty LNPs and the STR-V003 groups.

**Figure 8:**
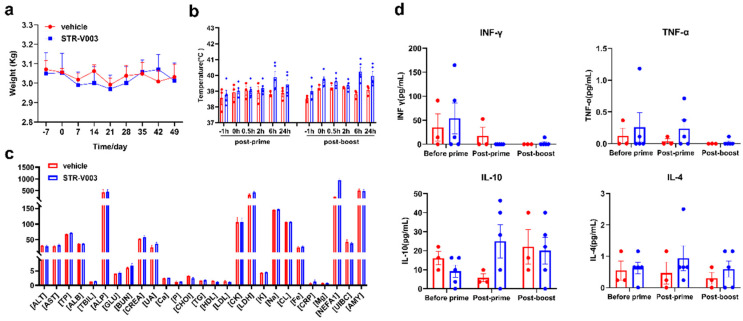
Well-tolerated STR-V003 in NHP. (**a**) Cynomolgus monkeys with two doses of the STR-V003 vaccine analogue on Day 0 and Day 21, respectively, with 300 ug of mRNA for each monkey. Then, the body weight and (**b**) body temperature of the monkeys were measured at different time points. (**c**) In addition, blood was collected within 24 h after the second vaccination to measure blood biochemical indexes. (**d**) Blood was collected within 24 h after each vaccination to measure the secretion amounts of different cytokines respectively. Data are shown as mean ± SEM.

In addition, the employment of a newly developed muscle-targeted lipid nanoparticle (LNP) in our experiments necessitated the conduction of simple safety data tests for this new vaccine in cynomolgus macaques. The parameters subjected to analysis included body weight (Figure 8a), body temperature (Figure 8b) and blood biochemistry (Figure 8c) after two dosages. It should be noted that cynomolgus macaques are semi-permissive for RSV replication, thereby precluding the performance of other pharmacodynamic evaluations specific to RSV.

As illustrated, no significant variations in body temperature and body weight were observed among the cynomolgus macaques subsequent to two administrations of STR-V003 analogue. Additionally, the blood biochemistry parameters, with particular emphasis on liver and kidney function markers, remained relatively unaltered after the administration process. There were almost no significant cytokine secretions, especially after post-boost treatment (Figure 8d). These results provide preliminary evidence suggesting that STR-V003 exhibits favorable performance with respect to these fundamental safety indices.

## 5. Discussion

In this study, STR-V003 is an mRNA vaccine produced by encapsulating the mRNA that encodes RSV preF protein in lipid nanoparticles (LNPs). A series of studies has been conducted to assess the immunogenicity and challenge protection of STR-V003.

The in vitro and in vivo pharmacology studies screened the mRNA sequences encoding RSV preF protein based on protein expression levels in transfected cell lines and immunogenicity in mice. The mRNA sequence (V003-210-T-plus) was determined to be the optimal sequence to be used in STR-V003.

Currently, there are no animal models that can fully recapitulate the pathogenesis of RSV infection in humans. The non-human primates (except chimpanzees) are only semi-permissive for hRSV replication and exhibit little or no clinical signs of disease when infected with hRSV. Even though cotton rats and mice are also only semi-permissive for hRSV, these two models have been informative in providing insights into RSV pathogenesis and mechanisms of immunity. These two models were, thus, selected to study RSV challenge protection by STR-V003. STR-V003 administered via IM injections on Day 1 and Day 21 prior to intranasal inoculation of RSV/A2 (in both models) or RSV/B9320 (in cotton rats) led to dose-dependent induction of RSV-neutralizing antibody (lasted for at least 7 weeks in cotton rats and 5 weeks in mice after first vaccination) and RSV preF-specific antibody (lasted for at least 4 weeks after first vaccination). A significant reduction in tissue viral loads was observed in both models. Both the immune responses and the protection induced by STR-V003 were stronger than those induced by FI-RSV. Importantly, unlike FI-RSV, STR-V003 did not cause enhanced lung pathology. Taken together, these results demonstrated that STR-V003 is able to induce robust and lasting immune responses and provide significant protection against RSV infection without inducing vaccine-enhanced disease (VED). It is noteworthy that STR-V003 vaccination induced both RSV A- and RSV B-neutralizing antibodies and reduced the viral loads of both RSV A and RSV B strains, suggesting that STR-V003 is capable of protecting infection of both RSV A and RSV B strains, the two major groups of RSV that cocirculate worldwide [30].

The safety studies did not identify unexpected toxicities following repeated IM injections of STR-V003 (50, 75 or 100 µg, Q2W, three administrations in total) in SD rats. The changes observed were considered to be the expected immune responses to the mRNA vaccine or secondary to inflammation. The local irritation at the injection sites observed in SD rats in safety studies was reversible, as toxicology studies indicated that STR-V003 did not affect vital physiological functions (e.g., central nervous system, respiratory and cardiovascular functions) other than the immune system.

## 6. Conclusions

A comprehensive panel of studies was conducted to evaluate the immunogenicity, protection efficacy and safety profiles of STR-V003. The above results showed that the test vaccine STR-V003 had good immunogenicity and in vivo antiviral efficacy had no disease enhancement effect. The scope of the toxicological evaluation of STR-V003 demonstrated an acceptable safety profile.

## Figures and Tables

**Table 1:** Empty LNPs/STR-V003-related microscopic findings, terminal necropsy (Day 32).

Sex	Male							Female						
Group	1	2	3	4	5	6	7	1	2	3	4	5	6	7
STR-V003/Empty LNPs		STR-V003			Empty LNPs				STR-V003			Empty LNPs		
Dose/Rat	0	1	1.5	2	1	1.5	2	0	1	1.5	2	1	1.5	2
Examined	5	5	5	5	5	5	5	5	5	5	5	5	5	5
**Thymus**														
Decreased, Lymphocytic Cortex; Medulla														
Minimal	0	0	0	1	1	0	0	0	1	0	0	0	0	1
Slight	0	2	5	2	0	5	0	0	0	3	2	3	5	4
Moderate	0	0	0	2	0	0	4	0	0	0	2	0	0	0
**Spleen**														
Increased, Lymphocytic White pulp														
Minimal	0	0	0	1	0	0	1	0	0	2	0	0	2	1
Slight	0	0	0	0	0	0	1	0	0	0	0	0	0	1
Increased, Extramedullary Hematopoiesis, Red Pulp														
Minimal	0	1	1	1	0	1	0	0	0	1	2	0	1	1
Slight	0	0	0	0	0	0	0	0	0	0	0	0	0	1
**Liver**														
Vacuolation, Hepatocyte, Portal area														
Minimal	0	1	2	2	2	2	3	1	4	3	1	1	1	0
Slight	0	0	1	2	1	3	2	0	0	1	0	2	3	2
Moderate	0	0	0	0	0	0	0	1	0	1	3	1	1	3

## Data Availability

The data presented in this study are available upon request from the corresponding author.

## Supplementary Materials

The following supporting information can be downloaded at: https://www.mdpi.com/article/10.3390/vaccines13060625/s1, Figure S1: In vivo results of DOE study; Figure S2: Analysis of In vivo results of DOE study; Figure S3: RSV F protein specific antigenic sites of V003-210-T-plus detected by FACS; Table S1: Size, PDI and EE of screening lipids; Table S2: Optimized formulation ratio of F11; Table S3: Histopathology Semiquantitative Scoring Criteria; Table S4: Grouping of Cotton Rats; Table S5: Pathology scores of lung tissues in cotton rats on Day 54; Table S6: Organ Weights Changes (Day 32).
