# Supplementary material for: Highly Effective mRNA-LNP Vaccine Against Respiratory Syncytial Virus (RSV) in Multiple Models
Source: Vaccines (Basel). 2025 Jun 10;13(6):625. doi: 10.3390/vaccines13060625 (PMC12197462; doi:10.3390/vaccines13060625)
Supplement: Supplementary file 1 [file vaccines-13-00625-s001.zip › vaccines-3694889-supplementary.pdf]

Highly effective mRNA-LNP vaccine against Respiratory Syncytial Virus (RSV) in multiple models

Huarong Bai †, Xueliang Yu †, Yue Gao, Qin Li, Baigang Wen and Rongkuan Hu *

Starna Therapeutics Co., Ltd., Suzhou 215123, China
* Correspondence: hurongkuan@starnatx.com
† These authors contributed equally to this work

Supplementary materials

Supplementary Figure S1. In vivo results of DOE study. Six-week-old male C57BL/6 mice, weighing about 20g, fed in the specific-pathogen free (SPF) feeding room were selected. The FLuc mRNA containing lipid particles prepared as described as in the main text were administered via intramuscular injection ("IM") at the dosage of 0.25 mg/kg. After 6 hours, 100μL of 30mg/mL D-luciferin (potassium salt) was intraperitoneally injected into each mouse. After 10 minutes, the total fluorescence intensity of each mouse was observed and recorded by in vivo imaging system. And the total fluorescence intensity (p/s) was recorded.

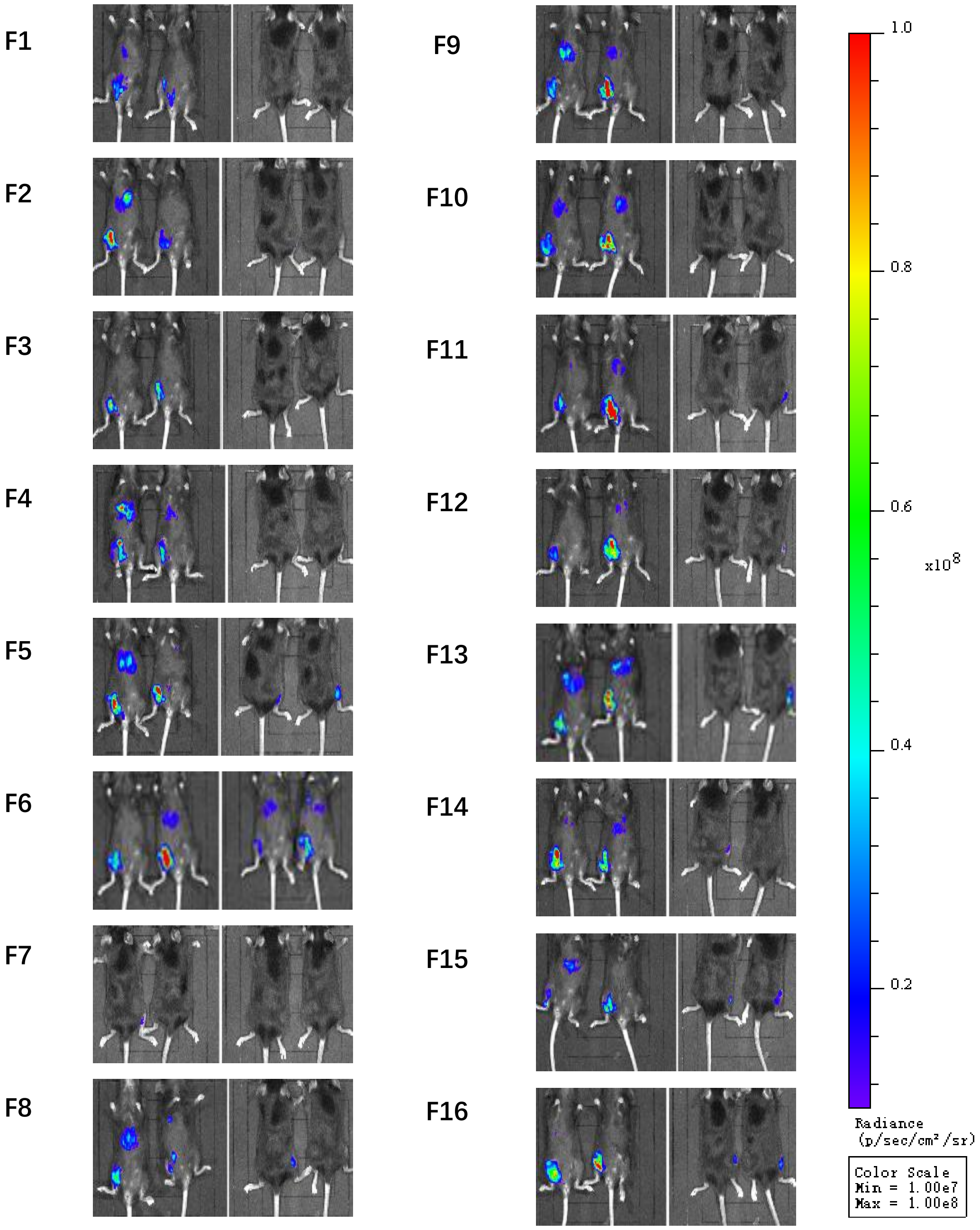

Supplementary Figure S2. Analysis of In vivo results of DOE study. y-axis means the average total fluorescence intensity (p/s), x-axis means the molar ratio (%) of each component.

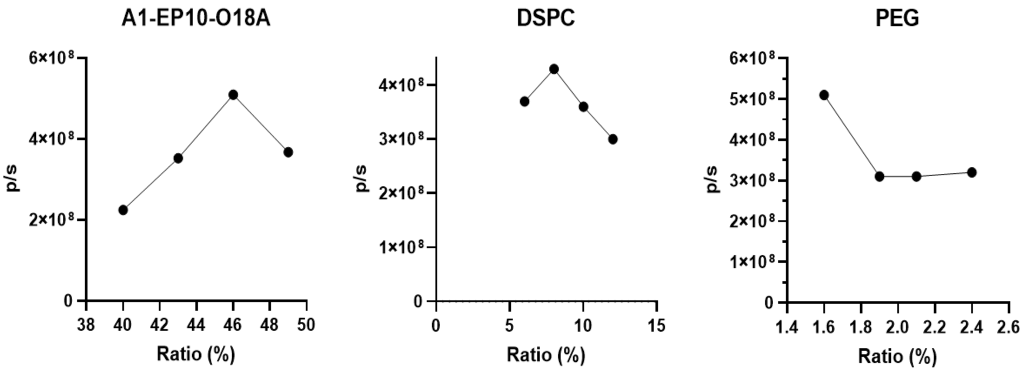

Supplementary Figure S3. RSV F protein specific antigenic sites of V003-210-T-plus detected by FACS. Antibodies recognizing antigenic site I, II, III, IV, V and Φ were used to stain RSV preF protein in HT1080 cells. Flow cytometric data were quantitatively evaluated using FlowJo software.

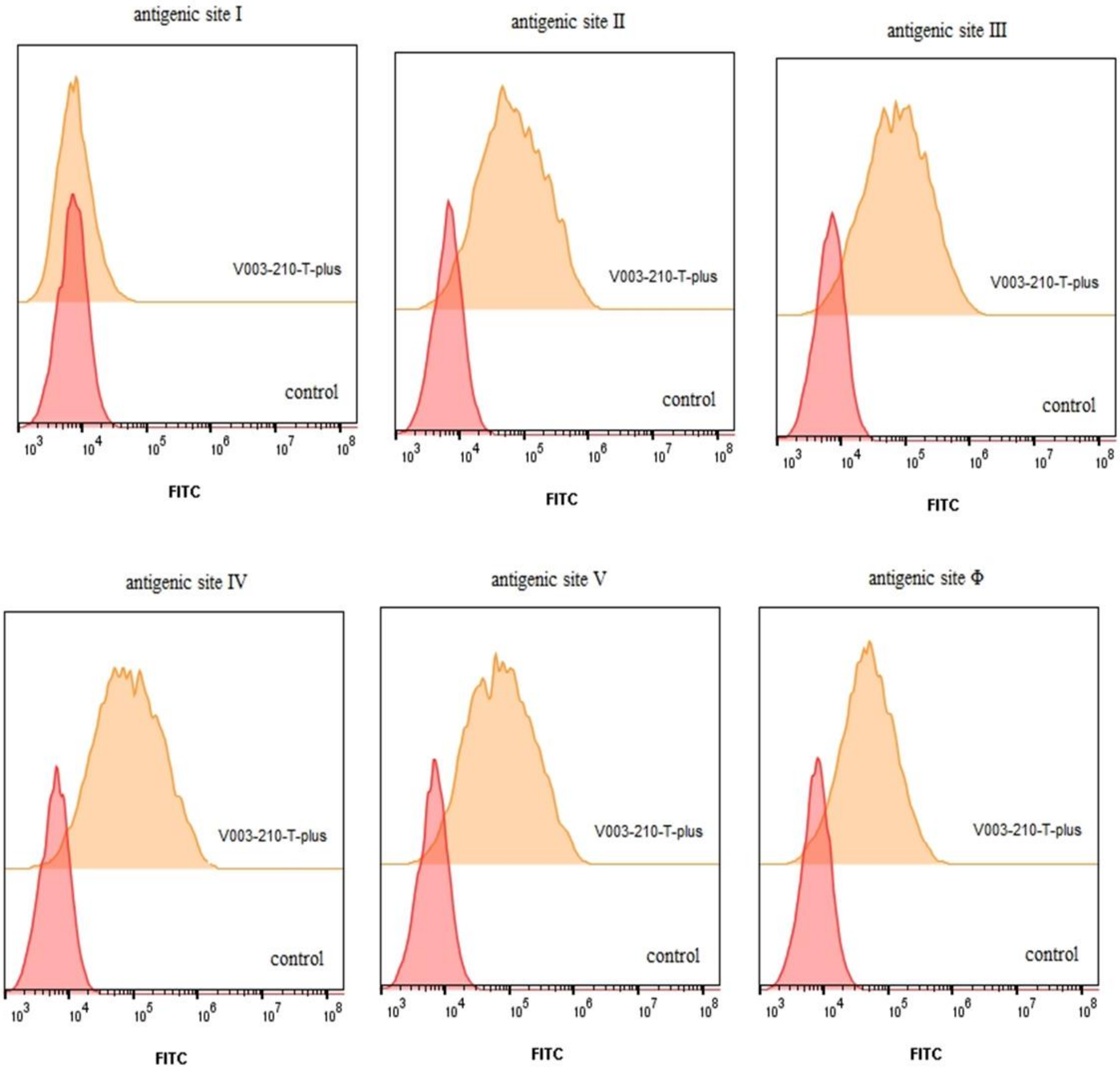

Supplementary Table S1. Size, PDI and EE of screening lipids.

Lipid	Size	PDI	EE
MC3	104.8	0.061	79.7%
A1-EPO15-O18B	111.9	0.152	95.3%
A1-EP10A-O18B	96.6	0.112	84.7%
A1-EP10-O18B	105.1	0.119	93.9%
A1-EPO15-O18A	104.6	0.148	92.3%
A1-EP10A-O18A	102.9	0.045	89.0%
A1-EP10-O18A	113.9	0.142	87.5%

Supplementary Table S2. Optimized formulation ratio of F11.

**Compound**	**Lipid**	**DSPC**	**Chol**	**PEG**
**Ratio(mol/mol)**	46.0	10.0	42.4	1.6

Supplementary Table S3. Histopathology Semiquantitative Scoring Criteria.

**Histological characterization**	**Score**	**Criteria**
alveolar area<br>Inflammatory infiltration of macrophages, lymphocytes and neutrophils in alveolar tissue	0	none or no visible lesion
	1	mild
	2	moderate
	3	marked
	4	severe
Perivascular<br>Inflammatory infiltration of macrophages, lymphocytes and neutrophils around blood vessels	0	none or no visible lesion
	1	mild
	2	moderate
	3	marked
	4	severe
Peribronchial<br>Inflammatory infiltration of macrophages, lymphocytes and neutrophils around the bronchioles	0	none or no visible lesion
	1	mild
	2	moderate
	3	marked
	4	severe
Interstitial<br>Inflammatory infiltration of macrophages, lymphocytes and neutrophils in the interstitium	0	none or no visible lesion
	1	mild
	2	moderate
	3	marked
	4	severe

Supplementary Table S4. Grouping of Cotton Rats.

**Group**	**Gender**	**Immunization Route**	**Immunizing Dose**	**Challenge Strain**
G1	3♂	IM	STR-V003 (5 μg)	RSV A2
G2	2♀ 1♂	IM	STR-V003 (20 μg)	RSV A2
G3	2♀ 1♂	IM	Vehicle	RSV A2
G4	1♀ 2♂	IM	STR-V003 (5 μg)	RSV B9320
G5	2♀ 1♂	IM	STR-V003 (20 μg)	RSV B9320

G6	2♀ 1♂	IM	Vehicle	RSV B9320

Supplementary Table S5. Pathology scores of lung tissues in cotton rats on Day 54.

Group	Histological characterization			
	Perivasculitis	Peribronchiolitis	Alveolitis	Interstitial pneumonia
G1 STR-V003 5ug/animal (RSV A2)	0	1	0	0
	1	1	0	0
	1	1	0	0
G2 STR-V003 20ug/animal (RSV A2)	0	1	0	1
	0	1	0	0
	0	1	0	0
G3 Vehicle (RSV A2)	0	0	0	0
	1	1	0	0
	0	1	0	0
G4 STR-V003 5ug/animal (RSV B9320)	0	2	0	0
	0	2	0	0
	0	2	0	0
G5 STR-V003 20ug/animal (RSV B9320)	0	1	0	0
	0	1	0	0
	0	2	0	0
G6 Vehicle (RSV B9320)	1	2	0	0
	1	2	0	0
	0	1	0	0

Supplementary Table S6. Organ Weights Changes (Day 32).

Parameter (% Change [a])		Negative Control	STR-V003 (dose/animal)			Empty LNPs (dose/animal)		
			1	1.5	2	1	1.5	2
M	**Thymus**							
	Organ Weights (g)	0.4916	-35.9%*	-40.7%*	-52.4%*	-28.4%*	-41.9%*	-52.8%*
	organ-to- brain weight ratios (%)	23.2654	-34.8%*	-37.6%*	-48.7%*	-25.7%	-39.7%*	-51.6%*

	organ-to-body weight ratios (%)	0.1102	-31.3%*	-32.6%*	-45.7%*	-22.3%	-36.3%*	-46.7%*
	**Spleen**							
	Organ Weights (g)	0.8326	+15.6%	+24.2%	+31.9%	+10.4%	+15.0%	+18.3%
	organ-to- brain weight ratios (%)	39.5158	+17.3%	+31.0%*	+42.1%*	+14.2%	+18.8%	+20.7%
	organ-to-body weight ratios (%)	0.1869	+24.9%	+39.9%*	+51.4%*	+19.5%	+27.3%	+34.1%*
**F**	**Thymus**							
	Organ Weights (g)	0.4630	-17.8%	-39.8%*	-42.8%*	-37.5%*	-36.2%*	-25.9%*
	organ-to- brain weight ratios (%)	23.8299	-19.0%	-38.1%*	-42.1%*	-36.2%*	-33.5%*	-27.0%*
	organ-to-body weight ratios (%)	0.1608	-13.7%	-34.8%*	-38.5%*	-32.1%*	-31.5%*	-23.9%
	**Spleen**							
	Organ Weights (g)	0.6440	+26.0%*	+22.3%*	+32.1%*	+17.8%	+18.9%*	+35.2%*
	organ-to- brain weight ratios (%)	33.1351	+23.9%*	+25.6%*	+34.3%*	+20.1%*	+23.8%*	+33.5%*

	organ-to-body weight ratios (%)	0.2236	+32.3%*	+32.9%*	+41.7%*	+28.8%*	+27.5%*	+38.8%*
